# Caspase-8: A Novel Target to Overcome Resistance to Chemotherapy in Glioblastoma

**DOI:** 10.3390/ijms19123798

**Published:** 2018-11-29

**Authors:** Giulia Fianco, Claudia Contadini, Alessandra Ferri, Claudia Cirotti, Venturina Stagni, Daniela Barilà

**Affiliations:** 1 Department of Biology, University of Rome “Tor Vergata”, 00133 Rome, Italy; giulia.fianco@gmail.com (G.F.); claudiacontadini@gmail.com (C.C.); alessandraferri4@gmail.com (A.F.); claudiacirotti89@gmail.com (C.C.); venturina.stagni@gmail.com (V.S.); 2 Laboratory of Cell Signaling, Istituto di Ricovero e Cura a Carattere Scientifico (IRCCS) Fondazione Santa Lucia, 00179 Rome, Italy

**Keywords:** Caspase-8, glioblastoma, FLIP, Src kinase, tyrosine phosphorylation, NF-κB, tumor microenvironment, chemotherapy and radiotherapy, DNA damage, apoptosis

## Abstract

Caspase-8 was originally identified as a central player of programmed cell death triggered by death receptor stimulation. In that context, its activity is tightly regulated through several mechanisms, with the best established being the expression of FLICE-like inhibitory protein (FLIP) family proteins and the Src-dependent phosphorylation of Caspase-8 on Tyr380. Loss of apoptotic signaling is a hallmark of cancer and indeed Caspase-8 expression is often lost in tumors. This event may account not only for cancer progression but also for cancer resistance to radiotherapy and chemotherapy. Intriguingly, other tumors, such as glioblastoma, preferentially retain Caspase-8 expression, and high levels of Caspase-8 expression may correlate with a worse prognosis, suggesting that in this context this protease loses its apoptotic activity and gains additional functions. Using different cellular systems, it has been clearly shown that in cancer Caspase-8 can exhibit non-canonical functions, including promotion of cell adhesion, migration, and DNA repair. Intriguingly, in glioblastoma models, Caspase-8 can promote NF-κB-dependent expression of several cytokines, angiogenesis, and in vitro and in vivo tumorigenesis. Overall, these observations suggest that some cancer cells may hijack Caspase-8 function which in turn promote cancer progression and resistance to therapy. Here we aim to highlight the multiple functions of Caspase-8 and to discuss whether the molecular mechanisms that modulate the balance between those functions may be targeted to dismantle the aberrant activity of Caspase-8 and to restore its canonical apoptotic functionality.

## 1. Introduction

Resistance to apoptosis is a hallmark of cancer and it has been clearly linked both to tumor development as well as to cancer resistance to therapy [1]. Consistently, its targeting has been proposed as a valuable strategy to ameliorate the therapeutic response [2].

Caspase-8 is a canonical cysteine protease required for the initiation and the execution of apoptosis; it is a key actor of death receptor-induced programmed cell death and its activation is absolutely required for the functionality of this pathway [3,4]. Consistently with its central role in death receptor-induced apoptosis [4], it is historically considered as a tumor suppressor. This is in agreement with the observation that Caspase-8 expression or its enzymatic activity, required for the execution of the apoptotic signal, are often impaired in cancer (reviewed in [5]). Indeed Caspase-8 is genetically or epigenetically silenced in several tumors such as neuroblastoma and medulloblastoma [6,7]. One puzzling issue arises from the observation that Caspase-8 expression is retained and even aberrantly overexpressed in some tumors, including hepatocellular carcinoma and glioblastoma (reviewed in [5]). For this reason, the work of many laboratories in the field has been aimed at two major objectives: (1) uncover the molecular mechanisms that allow cancer cells to be resistant to apoptosis even in the presence of high levels of Caspase-8 expression; (2) uncover novel non-canonical functions of Caspase-8 and elucidate their role in cancer development and therapy.

It has been clearly shown that in several tumors Caspase-8 expression is retained but its enzymatic activity is impaired by the aberrant overexpression of FLICE-like inhibitory protein (FLIP) proteins, the main modulators of Caspase-8 activation (reviewed in [8,9]). In addition, post-translational modifications, including tyrosine phosphorylation and ubiquitination have been identified as balancers of the cascade of events that lead to the enzymatic activation of Caspase-8 [10].

In the last 15 years the list of non-canonical functions of Caspase-8 has lengthened and it includes cell death related functions, such as autophagy and necroptosis (reviewed in [11]) as well as cell death unrelated functions, such as the modulation of NF-κB activity and of the development of the immune system (reviewed in [11,12]) and the promotion of cell adhesion and migration in cancer cells [13,14].

Overall these findings suggest the hypothesis that, in some conditions, tumors may rewire Caspase-8 function from the canonical apoptotic pathway to other signaling cascades that may support tumorigenesis and also trigger resistance to radio and chemotherapy.

Glioblastoma is a very aggressive tumor, characterized by a median survival from initial diagnosis of less than 15 months and an average 2-year survival rate of 26–33% [15]. Therefore, the identification of novel more effective therapeutic strategies is urgently needed. Interestingly, Caspase-8 expression in glioblastoma is often retained (reviewed in [5]), and in particular Caspase-8 is upregulated in the mesenchymal subgroup [16]. We have recently shown that in glioblastoma cells lines Caspase-8 can sustain neoplastic transformation in vitro and tumor growth in vivo [17,18]. Importantly, it promotes NF-κB activity and sustains cytokine production and neoangiogenesis. Furthermore, its silencing may sensitize cancer cells to temozolomide treatment [17,18].

This review summarizes the current knowledge of Caspase-8 canonical and non-canonical functions and on the molecular mechanisms that allow the modulation of Caspase-8 enzymatic activity with particular emphasis on its major regulators including FLIP proteins, Caspase-10 and post-translational modifications. Moreover, we will discuss how the different functions of Caspase-8 may impinge on the therapeutic response and how the molecular mechanisms that modulate their switch-on and -off may be exploited to ameliorate the response of glioblastoma to chemotherapy and radiotherapy.

## 2. Caspase-8 Structure and Enzymatic Function

Human Caspase-8 is a canonical cysteine protease encoded by *CASP8* gene located on chromosome 2q33-34. There are eight different isoforms, Caspase-8a-h, of which Caspase-8a and -8b are predominantly expressed and easily detectable. Caspase-8a is considered the “canonical” form and for the remainder of this text its sequence will be used as a reference (reviewed in [5,11])

Procaspase-8 is structured in a N-terminal prodomain which contains two Death Effector Domains (DED1 and DED2), responsible for sensing stimuli and activate the zymogen, and a C-terminal domain containing a large (p20/p18) and a small domain (p12/10) and a short linker region between them. Structural studies have shown that the large subunit contains the active catalytic cysteine while the small subunit is the substrate binding region, upon death receptor stimulation [19,20].

To be activated, the inactive monomeric Procaspase-8 requires a cascade of events to form the catalytically active Caspase-8. Overall, the autoprocessing of the zymogen allows the release of the large and small subunits and the assembly of the active heterotetramer made by two large and two small subunits [19,20].

## 3. Caspase-8 Cell Death Related Functions

### 3.1. Caspase-8 as a Central Transducer of Death Receptor-Induced Apoptosis

Caspase-8 has been originally identified as an essential player of death receptor-induced apoptosis (extrinsic apoptosis) [3]. In this context, Caspase-8 is activated following the formation of the Death-Inducing Signaling Complex (DISC) [21], a multiprotein complex made by a death receptor, the adapter protein FADD (Fas-Associated with Death Domain) and Procaspase-8 [3].

Extrinsic apoptosis starts when a death ligand (i.e., FasL, TNF-Related Apoptosis-Inducing Ligand) (TRAIL) binds a death receptor (i.e., Fas, TNF-related apoptosis-inducing ligand-receptor).

(TRAIL-R) on the cell surface causing a conformational change responsible for the exposure of a death domain (DD) on the cytoplasmic side, recognized by the C-terminal DD domain of FADD adaptor protein [22]. Once FADD is recruited to the death receptor, a conformational change makes its N-terminal DED domain able to recruit Procaspase-8 by its DED1 domain, forming the basic structure of the DISC complex. Additional Procaspase-8 molecules bind with their DED1 domain to the still free DED2 domain of the former Procaspase-8 molecule leading to DED-dependent Procaspase-8 unidirectional oligomerization [23].

The formation of oligomeric platforms makes much more favorable the dimerization of mature Caspase-8, process that is unlikely under normal circumstances [24]. Indeed, monomeric Procaspase-8 must dimerize to become active [25], according to the “induced proximity” model [3].

Two consecutive autoproteolytic cuts are responsible for dimerization: the first cleavage site is in the linker region (D210 and D216) and it partially activates the protein, while the second is in the linker region (D374 and D384) between the prodomain and the large subunits; this event fully activates Caspase-8 and allows the formation of the mature tetrameric complex released from the DISC [25,26].

Mature and active Caspase-8 can cleave several enzymatic and structural proteins therefore executing the apoptotic pathway. Importantly, effector Caspases-3 and -7, normally present in quiescent form as dimeric zymogens are activated by Caspase-8-dependent cleavage; moreover Caspase-8 cleaves BID (BH3 Interacting-domain Death agonist), a BH3 domain containing protein belonging to the BCL-2 (B-Cell Lymphoma 2) family, whose cleaved fragment (cBID) activates BAX (Bcl-2 Associated X protein) and BAK (Bcl-2 homologous Antagonist Killer) apoptotic proteins, promoting mitochondrial outer membrane permeabilization (MOMP), the subsequent release of apoptotic proteins from the mitochondria to the cytosol and apoptotic cell death [27].

### 3.2. Caspase-8 in Other Programs of Death

The work of several laboratories identified a role for Caspase-8 also in other programs of death involving necroptosis and autophagy, suggesting that Caspase-8 activity may modulate the balance between autophagy, apoptosis and necroptosis [11].

Caspase-8 genetic or pharmacological inhibition in mammalian cells activated cell death depending on autophagy. Therefore, endogenous basal activity of Caspase-8 seems to play a prosurvival function [28]. More recently, the last step of autophagosome formation has been identified as a novel target to switch cytoprotective autophagy to apoptosis through the non-canonical activation of Caspase-8 on immature autophagosomal membranes [29]. Modulation of Caspase-8 may therefore contribute to redirect cytoprotective autophagy to cell death in cancer cells that frequently display high autophagic flux and resistance to canonical apoptotic signaling [29].

In vivo studies have clearly established that the primary role for Caspase-8 in development is to inhibit necroptosis [11]. Disruption of Caspase-8 or FADD [30] expression leads to embryonic lethality in mice [31] due to the activation of necroptosis. This death pathway relies on the activation of RIPK1 (Receptor-Interacting serine/threonine-Protein Kinase 1) and RIPK3 (Receptor-interacting serine/threonine-protein kinase 3) kinases that are normally shut off by Caspase-8 activity that directly targets them. Remarkably, *casp8*^−/−^*ripk3*^−/−^ or *fadd*^−/−^*ripk1*^−/−^ double mutant mice are viable [32,33,34].

## 4. Caspase-8 Cell Death Unrelated Functions

### 4.1. Caspase-8 Modulates Cell Adhesion and Migration

Several studies have clearly highlighted the occurrence of an important crosstalk between Caspase-8 and the cytoskeleton remodeling that plays a crucial role in the modulation of cell adhesion and of cell migration. This issue has been extensively and recently reviewed with particular attention to its function in cancer progression and in metastasis development [13,14].

Caspase-8 subcellular localization has been reported to be modulated by unligated integrins which promote Caspase-8 translocation to cellular membranes; this event may contribute to the Integrin Mediated Death (IMD) [35]. In this regard Caspase-8 is often lost in neuroblastoma and this event accounts for the impairment of IMD and promotes metastatization [36]. Moreover, under non-apoptotic conditions, such as Caspase-3 deficiency, Caspase-8 may stimulate cancer cell migration and metastatization by enhancing Calpain activity [37], by interacting with focal adhesion [38] and with Rab5 [39]. This effect has been shown to be independent of Caspase-8 enzymatic activity and to rely more on its ability to act as a scaffold that allows the assembly of specific protein complexes. In this regard a crucial role is played by the aberrant activation of Src kinase and other Src family members in tumors which in turn may directly phosphorylate Caspase-8 on Tyr 380, as discussed in the next sections (reviewed in [14]).

### 4.2. Caspase-8 Modulates the Immune System and NF-κB Activity

The involvement of Caspase-8 in the proliferation and homeostasis of human immune cells was initially suggested by the observation of the immune system of individuals with an inherited Caspase-8 deficiency. These individuals showed defects in the activation of their T, B lymphocytes, and natural killer (NK) cells proving that Caspase-8 deficiency in humans is compatible with normal development, differently from mice, but it leads to immunodeficiency [40]. Consistently, additional studies have shown that selective impairment of Caspase-8 expression in T-cells in mice leads to immunodeficiency as well [41]. More recently, a role for Caspase-8 in macrophage differentiation and activity has also been reported (reviewed in [12]).

The molecular mechanisms that allow Caspase-8 to play such a central role in the immune system have not been fully elucidated yet. An important issue is the ability of Caspase-8 expression to promote NF-κB nuclear translocation and NF-κB-dependent transcription in several cellular systems. The first evidence was that Caspase-8 activates NF-κB through its N-terminal prodomain, independently of Caspase-8 enzymatic activity [42]. This finding was further supported by an independent study showing that Caspase-8 promotes NF-κB activation downstream TNFR (Tumor Necrosis Factor Receptor) stimulation [43].

Later on evidence for Caspase-8-dependent activation of NF-κB in B and T-cells have been produced [44,45]. Importantly NF-κB activation may promote FLIP protein accumulation [46] and FLIP promotes NF-κB activation as well [47].

We have recently shown that in glioblastoma cell lines Caspase-8 expression sustains NF-κB nuclear accumulation and correlates with NF-κB transcriptional activation of several cytokines such ad IL-6, IL-8, VEGF (Vascular Endothelial Growth Factor), IL-1 and βα [18]. Caspase-8 in glioma sustains cytokine production and angiogenesis and tumorigenicity in vivo, through pathways that have been only partially elucidated [18]. Overall these data point to Caspase-8 as a modulator of NF-κB activity and provide evidence for a novel function of Caspase-8 as a modulator of tumor microenvironment.

## 5. Molecular Mechanisms That Modulate Caspase-8 Enzymatic Activity and May Drive the Switch from Apoptotic to Non-Apoptotic Functions

According to its central role in the extrinsic apoptotic pathway Caspase-8 expression and activation are tightly regulated and controlled through multiple mechanisms that have been only partially elucidated. These pathways impinge on Caspase-8 enzymatic activity and/or may trigger the switch between its apoptotic and its non-apoptotic functions.

### 5.1. Caspase-8 Modulation by FLIP Proteins and by Caspase-10

FLIP family proteins are the main modulators of Caspase-8 activation downstream death receptor stimulation. These proteins share with Caspase-8 the presence in their N-terminal domain of two DED domains, and may therefore compete with Caspase-8 for the recruitment to the DISC (reviewed in [8,9]). Moreover, FLIP proteins can heterodimerize with Caspase-8, therefore impinging on its activation and on its functionality (reviewed in [8,9]).

In human three variants of FLIP proteins have been identified named FLIP-L (Long), FLIP-S (Short) and FLIP-R, while in mouse only the first two isoforms (FLIP-L and FLIP-S) have been identified (reviewed in [9]). While the expression FLIP-S has been clearly associated with the inhibition of Caspase-8 activation downstream death receptors, the expression of FLIP-L may have a dual role depending on the specific context and on its level of expression (reviewed in [8,9]).

Recent reports from several labs demonstrate that FLIP proteins may affect not only the apoptotic activity of Caspase-8 but also its role in necroptosis and autophagy [11]. The ability of FLIP to modulate Caspase-8 enzymatic activity and to modulate its recruitment to different signaling pathways, identifies FLIP as an intriguing switch for Caspase-8 functionality. Consistently, FLIP expression is often aberrantly upregulated in cancer [9]. This is in line with the observation that many tumors retain Caspase-8 expression, although its apoptotic activity is impaired by aberrant levels of FLIP proteins [9]. The upregulation of FLIP has been identified in several immunological tumors [48,49] and later on also in solid tumors (reviewed in [9]). In most cases the aberrant expression correlates with poor prognosis, most likely due to the impairment of the apoptotic cascade [9,50]. FLIP upregulation can interfere with sensitivity to chemo and radio therapy, impinging on the activation of the caspase-cascade in several tumors, as well as with Caspase-8 dependent induction of apoptosis triggered by death receptor stimulation: Furthermore, FLIP aberrant expression contributes also to cancer cell evasion from the immune system control [9,50]. 

Caspase-10 is a close homolog of Caspase-8, highly conserved throughout evolution, although absent in rodents [51,52]. Its endogenous expression has been well characterized in lymphoid cancer cell lines where Caspase-10 can be recruited to the DISC complex, similarly to Caspase-8, although it neither affects Caspase-8 activation nor it can compensate for Caspase-8 deficiency [53]. Recent works suggest that in other systems, Caspase-10 may play a more complex role in the modulation of Caspase-8 functionality. It negatively modulates Caspase-8 activation in response to death receptor stimulation in HeLa cells, promoting the activation of the prosurvival NF-κB-dependent cascade [54]. Moreover, in cancer cells, Caspase-10 expression is modulated by DNA damaging drugs and in turn may drive different responses to chemotherapy [55].

### 5.2. Caspase-8 Modulation by Post-Translational Modifications

#### 5.2.1. Phosphorylation

Caspase-8 functionality is also highly modulated by post-translational modifications, among which the most well characterized so far is phosphorylation [10]. The first phosphorylation has been mapped on human Casapase-8 protein on Tyr380; this residue is in the linker region between the large and the small catalytic subunits. This linker is a stretch of eleven residues present in the procaspase-8 protein, flanked by two Aspartic residues (D374 and D384) that undergo autoproteolytic cleavage during Caspase-8 activation and allow the release of the large and small subunit and the assembly of the fully active Caspase-8 tetramer. Tyrosine phosphorylation on Tyr380 is dependent on Src family kinases [10]; this event affects Caspase-8 function in multiple ways. First of all, it interferes with the autoproteolytic cleavages and impairs Caspase-8 full activation downstream death receptor stimulation [10]. Consistently, phosphorylation on Tyr380 impairs the functionality of the death-inducing signaling complex therefore impinging on death receptor-induced apoptosis [56]. Moreover, the tyrosine phosphorylation allows the interaction with several SH2 (Src Homology 2) domain containing proteins, including p85α subunit of PI3K, the adaptor protein CrkL, p120RAC-GAP, SHP2, PLCγ and SHC (SH2-containing) and therefore promoting the subsequent recruitment of Caspase-8 to multiprotein complexes involved in cytoskeletal remodeling and in cell adhesion and migration (reviewed in [14]).

**Figure 1:**
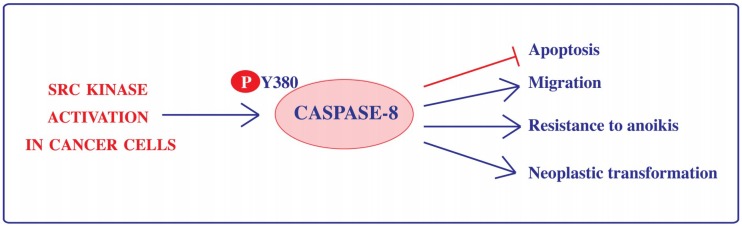
Src kinase-dependent phosphorylation on Tyr380 rewires Caspase-8 functionality in cancer cells. Phosphorylation on Tyr380 (P-Y380) impairs the apoptotic function of Caspase-8 and promotes its ability to sustain cell adhesion and migration, neoplastic transformation and to impair anoikis. Arrows indicate induction and T-bar indicates inhibition.

Importantly, Src kinase activity is aberrantly upregulated in many tumors [57]; consistently, the phosphorylation of Caspase-8 on Tyr380 has been reported in several cancer cell lines (including colorectal carcinoma, hepatocellular carcinoma and glioblastoma cell lines) and its occurrence has been correlated with impairment of apoptotic signaling downstream death receptor stimulation and resistance to anoikis [10,17]. Recently, the work of several laboratories clearly established that the phosphorylation of Tyr380 ensures not only the downregulation of Caspase-8 proapoptotic function but perfectly matches with the gain of new tumorigenic functions, including enhanced adhesion and migration properties ([37,38,39,58,59,60,61] in vitro neoplastic transformation, and resistance to anoikis [17] (Figure 1)).

Whether Tyr380 phosphorylation may play a role in the interaction of Caspase-8 with FLIP or with Caspase-10, or whether it may modulate Caspase-8 ability to promote NF-κB activation or to modulate autophagy and necroptosis, is still unknown.

It is important to point out that Tyr380, although conserved throughout evolution, is not present in mice. In addition, Tyr380 Caspase-8 has been reported to be phosphorylated on other residues, including Tyr334 and Tyr448 located along the dimerization interface and serine and threonine residues (S256, S305, S347, T273). Future studies will further elucidate the significance of these modifications (reviewed in [14]).

#### 5.2.2. Ubiquitination

The apoptotic activation of Caspase-8 downstream death receptor stimulation is also modulated by ubiquitination. It has been reported that Cullin 3 ubiquitin ligase may be recruited to the DISC complex and promote Caspase-8 polyubiquitination and full enzymatic activation [62]. Conversely, TRAF2 (TNF Receptor Associated Factor 2) ubiquitin ligase promotes Caspase-8 polyubiquitination on K48 which targets the protein for proteasomal degradation therefore switching off the apoptotic signal [63].

Future studies will clarify whether these pathways may be deregulated in cancer and whether their modulation may be exploited in cancer therapy.

## 6. Caspase-8 in Glioblastoma

### 6.1. Caspase-8 Expression and Function

The finding that Caspase-8 expression is retained in many tumors, including hepatocellular carcinoma and some glioblastoma, suggests that in these contexts its apoptotic activity may be switch off and its function rewired, as described in the previous section, to sustain tumor growth (reviewed in [5]).

Glioblastoma is a malignant type of central nervous system tumor. The unfavorable prognosis is mainly linked to NF-κB aberrant activation [64], to the extensive angiogenesis associated with this tumor [65] and to its ability to infiltrate throughout the brain tissue and to resist to chemotherapy [15].

Several studies provided evidence for very heterogeneous levels of Caspase-8 expression in glioblastoma cell lines as well as in primary tumors [5,16,66,67].

We have recently reported that Caspase-8 expression is retained in U87MG and U251MG glioblastoma cell lines and that in these model systems it may promote neoplastic transformation, resistance to anoikis and to therapeutic treatment with temozolomide in vitro [17,18]; furthermore, it sustains NF-κB activation, cytokine production, neoangiogenesis and tumor growth in vitro and in vivo [18]. Importantly, this observation is in agreement with large-scale expression studies that revealed Caspase-8 upregulation in glioblastoma patients compared to normal tissue, in particular in the mesenchymal subtype [16]. Bioinformatic analysis of clinical data derived from glioblastoma patients identify a strong correlation between high levels of Caspase-8 expression and worse prognosis [18] further confirming the central role of Caspase-8 expression both in the development and in the response to therapy.

Importantly, Src kinase activity is often aberrantly upregulated in glioblastoma downstream mutations that upregulate EGFR (Epidermal Growth Factor Receptor) or other receptor tyrosine kinases which in turn may drive the constitutive activation of Src; indeed Src has been identified as a potential target for glioblastoma therapy [68]. We could show that Src promotes Caspase-8 phosphorylation on Tyr380 in U87MG and U251MG glioblastoma cell lines, and this event contributes to their neoplastic transformation and resistance to anoikis [17].

### 6.2. Caspase-8 and Glioblastoma Therapeutic Treatment

Glioblastoma is a very aggressive tumor. Its treatment includes maximum safe surgical resection, followed by radiotherapy plus concomitant and maintenance temozolomide chemotherapy. Unfortunately, almost all patients experience tumor progression. Several laboratories are therefore looking for novel therapeutic approaches and a large number of clinical trials are currently ongoing in order to ameliorate the prognosis of glioblastoma patients [15]. Radiotherapy and chemotherapy target cancer cells by inducing DNA damage, which in turn may drive cell death. Apoptosis is a major cell death pathway and indeed evasion from apoptosis is one of the most prominent theories that explain cancer cell resistance to therapeutic strategies such as radio- and chemo-therapy, which trigger the DNA damage response. This issue provides the rationale behind the design of targeting the cell death machinery to improve the efficacy of anticancer therapies [2,69].

The central role of Caspase-8 in apoptosis suggests that its loss of expression or the impairment of its apoptotic activation may trigger cancer cell resistance to therapeutic approaches that relay on the apoptotic cascade. Consistently, the levels of FLIP proteins, the main modulators of Caspase-8 enzymatic activation, are dramatically upregulated in several cancers and correlate with a poorer clinical outcome, likely related to FLIP’s cell death inhibitory function on Caspase-8 [9]. Indeed, tumors with low Caspase-8, or high levels of FLIP, may inherently be resistant to such therapies (reviewed in [9,50]) The biological manipulation of the expression of Caspase-8 or of those proteins that may modulate its function such as FLIP, may therefore synergize with radio- and chemotherapy to enhance tumor killing [5]. It is intriguing to highlight that DNA damaging agents may directly impinge on Caspase-8 apoptotic activation [2]. As an example, ATM kinase, one of the central proteins involved in the DNA damage response induced by radio and chemotherapeutic drugs, is directly involved in the regulation of Caspase-8 functionality [70,71]. Chemotherapeutic agents that trigger DNA damage, drive ATM activation which in turn sustains the ubiquitination and degradation of FLIP proteins and Caspase-8 enzymatic activation [70,71].

Death receptor stimulation induced apoptosis may also represent a complementary strategy to implement cancer therapy. In particular, TRAIL receptor stimulation has been considered as a promising approach because of its selectivity for cancer cells (reviewed in [67,72]). In agreement with the central role of Caspase-8 in this pathway, several studies highlight that TRAIL sensitivity in glioblastoma is tightly dependent on the expression levels of Caspase-8 or on the expression levels or activity of the modulators of its apoptotic activation such as FLIP proteins ([67,73]). Therefore, strategies that may modulate the expression of the apoptotic activation of Caspase-8 represent a valuable approach to ameliorate the response to this therapeutic approach. In this regard it is intriguing to notice that radio and chemotherapy treatments may enhance TRAIL sensitivity most likely through the modulation of FLIP protein levels (reviewed in [74]).

As already pointed out, the expression of Caspase-8 is very heterogeneous in different glioblastoma cell lines as well as in different glioblastoma primary tumors. This may explain the different sensitivity in vitro and in vivo to radiotherapy, chemotherapy, and TRAIL treatment. Future studies will also clarify whether, strategies to target FLIP protein expression, to impair Caspase-8 phosphorylation on Tyr380, or to impinge on Caspase-10 expression may be beneficial in glioblastoma. In particular, since Src kinase activity is aberrantly upregulated in many glioblastoma and can trigger Caspase-8 phosphorylation in these contexts, cotreatment with Src kinase inhibitors, such as dasatinib, already in clinical trials, may represent a valuable approach to enhance Caspase-8 apoptotic activation in response to radio and chemotherapy as well as to TRAIL receptor stimulation.

As discussed above, standard chemotherapy regimens promote DNA damage and are typically thought to rely on the canonical apoptotic activity of Caspase-8 and on apoptosis for the elimination of cancer cells. However, the role of Caspase-8 in the DNA damage response has not been fully elucidated yet. In this regard, a recent work identified an emerging non-canonical role of Caspase-8 in the DNA damage response that deserves further consideration. Using as model system hepatocellular carcinoma cells, the authors demonstrate that Caspase-8 expression is required for the phosphorylation of H2AX in response to DNA damage induction, which is important to initiate DNA repair. Mice deficient for Caspase-8 lose the capacity to phosphorylate H2AX in response to treatment with the chemotherapeutic drug doxorubicin [75]. H2AX phosphorylation does not require Caspase enzymatic activity, as it is retained in the presence of a pan-caspase inhibitor, as well as mice that express the uncleavable and therefore inactive mutant Caspase-8-D387A [75]. c-FLIP, FADD, RIPK1 and RIPK3 inhibition phenocopy Caspase-8 deletion, suggesting that Caspase-8 may be part of a multiprotein complex that assists to H2AX phosphorylation [75]. Although no results on glioblastoma cellular models have been reported yet, this pathway is conserved in other cancer cell types, demonstrating that Caspase-8 contributes to the functionality of the DNA damage response and its loss therefore promotes genomic instability and tumor development. Intriguingly, this report suggests that the inhibition of Caspase-8, although detrimental for apoptosis induction, may enhance the sensitivity of cancer cells to DNA damaging agents, most likely independent of apoptosis, and may therefore represent a valuable therapeutic strategy [75]. The possible role of non-apoptotic cell death pathways in the modulation of cancer sensitivity to therapy deserves further elucidation as well.

Finally, the identification of several additional non-canonical functions of Caspase-8 along with the observation that its expression is surprisingly enriched in some glioblastoma [5,16] suggest a more complex role for Caspase-8 not only in cancer development but also in the modulation of chemotherapeutic sensitivity. As an example, the identification of Caspase-8 as a novel modulator of tumor microenvironment [18], suggests that the modulation of Caspase-8 may affect chemotherapy sensitivity not only by impinging on the apoptotic response but also through additional pathways. Therefore, while downregulation of Caspase-8 in glioblastoma may trigger resistance to apoptosis, it also correlates with a reduction of the level of expression of inflammatory cytokines; indeed the downregulation of Caspase-8 expression in this context results in an increased sensitivity to temozolomide through a still unknown molecular pathway [18].

## 7. Conclusions

**Figure 2:**
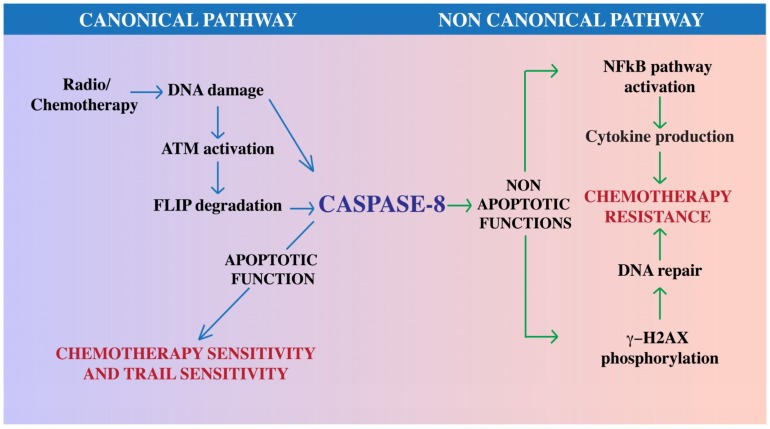
Role of Caspase-8 in cancer therapy. Caspase-8 may modulate the response to therapeutic approaches through canonical and non-canonical functions. Chemotherapy and radiotherapy promote DNA damage that in turn switches-on the enzymatic activation of Caspase-8, either directly or via the ATM-dependent downregulation of FLIP protein levels. Caspase-8 enzymatic activation promotes apoptosis therefore enhancing cancer cell sensitivity to chemotherapy and TRAIL. Cancer cells may rewire Caspase-8 functionality; in these contexts, Caspase-8 can promote NF-κB activity, cytokine production and DNA repair, therefore promoting resistance to chemotherapy. Overall, we suggest that this dual role of Caspase-8 in cancer may be exploited to ameliorate cancer therapy. Blue arrows for canonical pathway; green arrows for non canonical pathway.

Emerging evidence suggests that the retention of Caspase-8 in glioblastoma may interfere with the sensitivity to radio and chemotherapeutic approaches through multiple pathways, including the improvement of the DNA damage repair and the activation of NF-κB and cytokine production (Figure 2). In addition, Caspase-8 pro-apoptotic activity may be impaired by several mechanism including the aberrant expression of FLIP (reviewed in [9]) and also its Src-dependent phosphorylation on Tyr380 [10], which in turn sustains in vitro transformation and resistance to anoikis [17]. Future experiments will clarify whether Tyr380 phosphorylation may also play a role in the modulation of other Caspase-8 functions in glioblastoma. In this regard, Caspase-8 may become a sort of “trojan horse” for those tumors that retain its expression: future studies aimed to unveil the molecular mechanisms to switch back on its apoptotic function and to uncouple it from its non-canonical tumorigenic functions will clarify this issue.
